# Features of the course of severe and critical COVID-19 in pregnant women: A prospective cross-sectional study

**DOI:** 10.18502/ijrm.v22i3.16167

**Published:** 2024-05-15

**Authors:** Gulbanu Ganikyzy Shaimerdenova, Gulzhan Narkenovna Abuova, Saltanat Kulbayeva Nalibekkyzy

**Affiliations:** South Kazakhstan Medical Academy, Shymkent, Kazakhstan.

**Keywords:** COVID-19, Pregnancy, Infection.

## Abstract

**Background:**

At the beginning of the Coronavirus disease 2019 (COVID-19) pandemic, studies showed that the risk of severe disease was higher in pregnant women.

**Objective:**

This study investigates the characteristics of severe and critical types of COVID-19 coronavirus infection in pregnant women.

**Materials and Methods:**

This prospective cross-sectional study compared the medical records of 120 pregnant women with severe and very severe COVID-19 treated at the Infectious Disease Center, Shymkent, Kazakhstan from December 2021 to May 2022. Factors such as time of hospital admission, hospitalization period, maternal comorbidities, age, pregnancy and postpartum complications, pregnancy outcomes, and treatment type were analyzed.

**Results:**

87 (72.5%) pregnant women with severe and 33 (27.5%) with critical type of COVID-19 were included. The following data were obtained when comparing the pregnancy parity of the subjects, depending on the gestational age: in 1–12 wk, the indicator was 3.75 ± 0.95; in 13–27 wk 3.00 (Q1-Q3: 2.00–4.00), in 28–40 wk 3.00 (Q1-Q3: 2.00–4.00). Severe COVID-19 coronavirus infection occurs in women with more than a third pregnancy (Me 3.00 [Q1-Q3: 2.00–4.00]).

**Conclusion:**

There is a risk of disease progression to severe and critical COVID-19 in pregnant women older than 33 yr of age and at 28–40 wk gestation. Early referral to a doctor in hospital, timely hospitalization, and initiated treatment reduces the risk of aggravation of the patient's condition and development of formidable complications.

## 1. Introduction

The 21 ${\;}^{\text{st}}$ century began with a fundamental revision of the epidemic and pandemic potential of betacoronaviruses, which required the immediate deployment of a system of control as deep as for influenza, starting from the natural reservoir-bats (chiroptera, microchiroptera) to the organization of preventive and anti-epidemic measures (1). As a result, by December 2019, 6 representatives of the *Coronaviridae* family were known to humankind among 40 viruses.

The coronavirus disease 2019 (COVID-19) epidemic led to the discovery of the 7 ${\;}^{\text{th}}$ human coronavirus (2, 3). According to statistics, as of October 17, 2022, there were 629,959,595 people with COVID-19 coronavirus infection worldwide, with 6,571,489 deaths. In the Republic of Kazakhstan, 139,287 were registered, with 13,692 deaths (4). At the same time, 38,149 cases were registered in the city of Shymkent, Kazakhstan of which 1646 were pregnant for 2020–2022; 537 in 2020, 892 in 2021, and 217 women in 2022 (5).

According to recent studies, the risk of progression to severe disease in pregnant women was higher than in non-pregnant women (6–8). It should be noted that in pregnant women, the factors for the transition to a severe form are concomitant diseases of the cardiovascular system (arterial hypertension, chronic heart failure, concomitant respiratory chronic diseases, fibrotic changes in the lungs); endocrinopathies (diabetes mellitus, metabolic syndrome), obesity (body mass index > 40), and others; immunodeficiency states (oncological, hematological diseases); other severe chronic diseases (chronic kidney disease, liver disease, etc.) (9). The age group from 33–42 yr and the period from 22–36 wk of pregnancy also aggravate the course of COVID-19 (10). Severe infection is observed in 15% of patients, critical in 5%, mild and moderate in 80% which, according to the World Health Organization, is close to population indicators (11).

We studied the frequency and spectrum of complications, as well as the outcomes of pregnant women, the timing of the onset of COVID-19 complications, timely seeking medical help, and the risk of developing critical forms in case of late treatment. We detected the early onset of complications and timely identification of risk groups for severe and critical courses for early diagnosis and prescribed treatment, in a timely manner. Therefore, this study aimed to investigate the patterns of coronavirus COVID-19 infection in pregnant women.

## 2. Materials and Methods

### Setting

This prospective cross-sectional study compared the medical records of 120 pregnant women with severe and very severe COVID-19 treated at the Infectious Disease Center, Shymkent, Kazakhstan from December 2021 to May 2022. Factors such as time of hospital admission, hospitalization period, maternal comorbidities, age, pregnancy and postpartum complications, pregnancy outcomes, and treatment type were analyzed.

Women aged from 18–49 yr entered the study during COVID-19 and were followed up for 9 months.

The severity criteria included dyspnoea with slight exertion, when talking, at rest, respiratory rate 23–30 per min, resting SpO ${\;}_{2}$ < 92% with room air breathing, and persistent lymphopenia.

### Participants

Pregnant women with confirmed, severe, and critical COVID-19 infection; receiving treatment in infectious diseases hospitals of Shymkent city in 2020–2021; those who were independent from race and nationality; and the women of reproductive age were included in the study. Any condition incompatible with the study treatment (pregnancy without severe and critical COVID-19); mild to moderate severity of COVID-19 in pregnant women, absence of pregnancy, women of non-reproductive age, receiving treatment in non-infectious hospitals in Shymkent city, Kazakhstan in 2020–2021 were excluded from the study.

### Sample size

A confidence level of 95% and a power of 80% were considered to determine the sample size. We used G.power software (version 3.1) and was designed for 120 patients.

### Outcome

There is a risk of disease progression to severe and critical COVID-19 in pregnant women older than 33 yr of age and at 28–40 wk gestation. Early referral to a doctor in hospital, timely hospitalization, and initiated treatment may reduce the risk of aggravation of the patient's condition and development of formidable complications.

### Ethical considerations

The Local Bioethical Committee of South Kazakhstan Medical Academy, Shymkent, Kazakhstan, approved the study proposal (Code: 044–65/) (12).

### Statistical analysis

The normality of the distribution was tested according to Kolmogorov-Smirnov with the correction of Lillifors and Shapiro-Wilk. Because all data showed normal and non-normal distributions, the mean, standard deviation, median confidence interval, and interquartile range were subsequently used (13). Categorical variables are presented as absolute numbers, percentages, and frequencies. A p < 0.05 was considered statistically significant. The obtained data were processed using the IBM SPSS Statistics program (version 26.0, SPSS Inc Chicago, IL, USA). Nominal variables were analyzed using the Mann-Whitney, Pearson's Chi-square, and Fisher's exact tests.

## 3. Results

**Figure 1:**
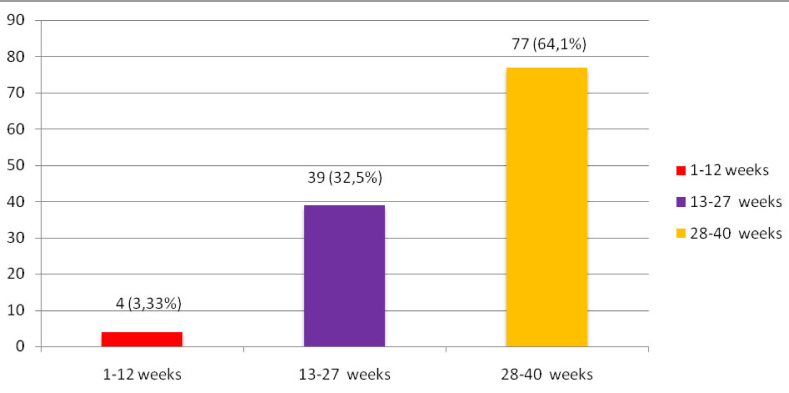
Distribution of patients with COVID-19 by gestational age.

The study included 87 (72.5%) women with severe COVID-19 and 33 (27.5%) pregnant women with a highly severe form of COVID-19. 4 (3.33%) women were in 1 ${\;}^{\text{st}}$ , 39 (32.5%) in 2 ${\;}^{\text{nd}}$ , and 77 (64.1%) were in the 3 ${\;}^{\text{rd}}$ trimester of pregnancy (Figure 1). Consequently, most women were hospitalized in the 3 ${\;}^{\text{rd}}$ trimester.

When comparing the birth parity of the subjects, depending on the gestational age we got next data: in 1–12 wk (3.75 ± 0.95). In the period of 13–27 wk 3.00 (Q1-Q3: 2.00–4.00), at 28–40 wk 3.00 (Q1-Q3: 2.00–4.00). Analysis of birth parity showed that the most common indications for hospitalization are the presence of 3 or more pregnancies. This confirms the availability of severe courses of COVID-19 in multiparous women.

We studied the following indicators, admission and hospitalization time, the number of comorbidities, age in admission time, complications of pregnancy and the postpartum period, pregnancy outcomes, and treatment (Table I).

When comparing patients with severe and critical forms of COVID-19, depending on the time of admission to the hospital, statistically significant differences were obtained (p = 0.05). Women with a critical form of COVID-19 infection were admitted to a medical facility later than pregnant women with a severe form. Analysis of the age of pregnant women showed statistically significant differences (p = 0.02) in severe form. The study found that pregnant women in the 18–25 age group had only 26 case of severe COVID-19 (26 [29.8]). Older age groups dominated the critical group. Older pregnant women are more likely to progress to a more severe form of COVID-19. The relationship between the compared features was medium (V = 0.270).

In the results of outcomes and pregnancy complications, no statistical significance (p = 0.06) was observed in the outcomes of the postpartum period. However, it should be noted that there were 18 cases of premature births. More than half of pregnancy complications were due to placental abruption, 17 cases. There were also 3 cases of intrauterine fetal death. During the study in the postpartum period, 6 cases of bleeding and 3 cases of sepsis were observed. A fatal case occurred in a pregnant woman with a highly severe form of COVID-19.

**Table 1:** Comparative table of indicators of pregnant women


**Index**		**Severe (n = 87)**	**Critical (n = 33)**	**P-value**
Terms of admission to the hospital (days)*		3 (2.0–3.0)	5 (2.5–7.0)	0.05#
Number of bed days (days)*		3 (7.0–10.0)	3 (7.50–11.5)	0.96
Number of comorbidities*		3 (2.50–4.50)	4 (3.50–5.00)	0.83
Age (yr)**				
	**18–25**	26 (29.8)	0	
	**26–32**	39 (44.9)	20 (60.6)	
	**33–42**	22 (25.3)	13 (39.4)	0.02#
Outcome of pregnancy**				
	**Prolongation**	56 (63.2)	20 (60.6)		
	**Preterm birth**	9 (11.1)	9 (29.7)	
	**Term delivery**	22 (24.8)	3 (9.09)	0.57
The complication in the postpartum period**				
	**Sepsis**	1 (12.5)	2 (60.6)		
	**Bleeding**	5 (62.5)	1 (19.7)	
	**Endometritis**	2 (25.0)	1 (19.7)	0.75
Complications of pregnancy**				
	**Intrauterine fetal death**	1 (3.3)	2 (60.6)		
	**Placental abruption**	16 (53.3)	1 (19.7)	
	**Preeclampsia**	12 (40)	1 (19.7)	
	**Chorioamnionitis**	1 (3.3)	0	0.72
The outcome of treatment**				
	**Improvement**	85 (97.4)	28 (84.8)		
	**Recovery**	2 (2.6)	0		
	**Refusal of treatment**	0	4 (12.1)	
	**Fatal outcome**	0	1 (3.1)	< 0.001#
*Data presented as Median (interquartile range), Mann-Whitney test, **Data were presented as n (%), Pearson's Chi-square and Fisher's exact test. ${\;}^{\#}$ Differences in indicators are statistically significant (p < 0.05)				

## 4. Discussion

The study included 87 (72.5%) women with a severe COVID-19 infection and 33 pregnant women with a highly severe form (27.5%). The following data were obtained, when comparing the pregnancy parity of the subjects, depending on the gestational age: in the 1–12 wk period, the indicator was 3.75 ± 0.95. In 13–27 wk 3.00 (Q1-Q3: 2.00–4.00), in 28–40 wk 3.00 (Q1-Q3: 2.00–4.00). The number of bed days spent in the hospital ranged from one to 23 days.

COVID-19 is an urgent problem around the world. Increased susceptibility to respiratory disease and severe pneumonia in pregnant women leads to severe hospitalization in intensive care units and mechanical ventilation (14–17). Pregnant women are characterized by a decrease in the immune response and other physiological changes during gestation, which is the cause of the above process. The severity of COVID-19 is associated with the entry of the virus into host cells after binding to angiotensin-converting enzyme 2. It should be noted that angiotensin-converting enzyme 2 replicates on cell membranes, which in turn has the property of being tropic to the placenta throughout the entire period of pregnancy. This phenomenon is a possible etiology of the susceptibility of pregnant women to COVID-19 (15–18). All these changes in a pregnant woman's body suggest a greater vulnerability to COVID-19 than other population categories.

It should be noted that most of the sick women in the gestation period of 28–40 wk. These patients is associated with an increasing load on the mother's body due to a sharp increase in the weight of the fetus. Increasing in the load on the cardiovascular and respiratory system of the mother and the needs rapidly growing fetus in this period of pregnancy. Multiparous patients were hospitalized more often than primiparas. Women with a severe form of COVID-19 were hospitalized later than pregnant women with a severe condition. Also, the older age group is a risk factor for transitioning to a more severe form of the disease. Comorbidities affect the prognosis of acute illness and an increased risk of severe symptoms. About 70% of patients requiring treatment in the intensive care unit have comorbidities (17–19). This was also confirmed in our study.

One limitation is that this study carried out in single center that does not provide extended results. Also the sample size was relatively small.

## 5. Conclusion

Thus, our study showed that a more severe coronavirus infection occurs in women with more than 3 pregnancies. In addition, pregnant women older than 33 yr and in 28–40 wk have a risk of transitioning to a severe form of the disease. Also, earlier treatment for inpatient care reduces the risk of aggravating the patient's condition.

##  Data availability

The data that support the findings of this study are available on a reasonable request from the corresponding author.

##  Author contributions

Gulbanu Ganikyzy Shaimerdenova: concept and design had full access to all of the data in the study and takes responsibility for the integrity of the data and the accuracy of the data analysis, statistical analysis, drafting of the manuscript, and supervision. Gulzhan Narkenovna Abuova: drafting of the manuscript, critical revision of the manuscript for important intellectual content. Saltanat Kulbayeva Nalibekkyzy: critical revision of the manuscript for important intellectual content, acquisition, analysis, or interpretation of data, and drafting of the manuscript. All authors approved the final manuscript and take responsibility for the integrity of the data.

##  Conflict of Interest

The authors declare that there is no conflict of interest.
